# Evolution of Optimized Hydride Transfer Reaction and Overall Enzyme Turnover in Human Dihydrofolate Reductase

**DOI:** 10.1021/acs.biochem.1c00558

**Published:** 2021-12-07

**Authors:** Jiayue Li, Jennifer Lin, Amnon Kohen, Priyanka Singh, Kevin Francis, Christopher M. Cheatum

**Affiliations:** † Department of Chemistry, University of Iowa, Iowa City, Iowa 52242, United States; ‡ Texas A&M University-Kingsville, Kingsville, Texas 78363, United States

## Abstract

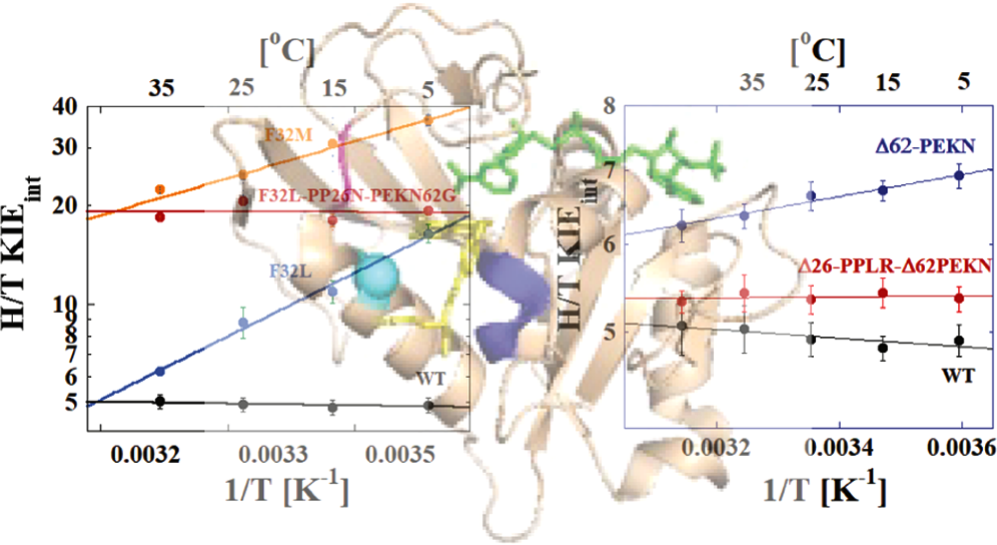

Evolution of dihydrofolate reductase (DHFR) has been studied using the enzyme from *Escherichia coli* DHFR (ecDHFR) as a model, but less studies have used the enzyme from *Homo sapiens* DHFR (hsDHFR). Each enzyme maintains a short and narrow distribution of hydride donor-acceptor distances (DAD) at the tunneling ready state (TRS). Evolution of the enzyme was previously studied in ecDHFR where three key sites were identified as important to the catalyzed reaction. The corresponding sites in hsDHFR are F28, 62-PEKN, and 26-PPLR. Each of these sites was studied here through the creation of mutant variants of the enzyme and measurements of the temperature dependence of the intrinsic kinetic isotope effects (KIEs) on the reaction. F28 is mutated first to M (F28M) and then to the L of the bacterial enzyme (F28L). The KIEs of the F28M variant are larger and more temperature-dependent than wild-type (WT), suggesting a broader and longer average DAD at the TRS. To more fully mimic ecDHFR, we also study a triple mutant of the human enzyme (F32L-PP26N-PEKN62G). Remarkably, the intrinsic KIEs, while larger in magnitude, are temperature-independent like the WT enzymes. We also construct deletion mutations of hsDHFR removing both the 62-PEKN and 26-PPLR sequences. The results mirror those described previously for insertion mutants of ecDHFR. Taken together, these results suggest a balancing act during DHFR evolution between achieving an optimal TRS for hydride transfer and preventing product inhibition arising from the different intercellular pools of NADPH and NADP^+^ in prokaryotic and eukaryotic cells.

## Introduction

The extraordinary rate accelerations achieved by enzymes have been studied extensively, but a comprehensive understanding of the physical principles governing enzyme catalysis has yet to be achieved. In particular, considerable debate exists over the possible roles of fast (fs to ps) protein motions in enzymatic reactions.^3−16^ Several computational, bioinformatic, and experimental studies have suggested a role for such motions in the hydride transfer reaction catalyzed by dihydrofolate reductase (DHFR; for reviews, see refs (3, 17)). The enzyme catalyzes the stereospecific transfer of the pro-*R* hydride from reduced nicotinamide adenine diphosphate (NADPH) to the C6 position of the dihydropterin ring of 7,8-dihydrofolate (DHF) to generate 5,6,7,8-tetrahydrofolate (THF). The reaction catalyzed by DHFR is essential in maintaining the intercellular pool of THF, which is required for the anabolism of purine nucleotides and some amino acids, thus making the enzyme an important drug target.

Experimental evidence regarding the role of active-site conformational sampling in the DHFR reaction has largely come from measurements of the temperature dependence of the intrinsic kinetic isotope effects (KIEs) on hydride transfer for the wild-type (WT)^18^ and mutant^19−24^ forms of the enzyme from *Escherichia coli* DHFR (ecDHFR). Theoretical studies have demonstrated that the magnitude and distribution of the hydride donor–acceptor distance (DAD) are the dominant factors in determining the temperature dependence of the KIEs.^5,7−9^ These studies support an interpretation in which the protein scaffold reorganizes the active site to achieve a DAD conducive for hydride tunneling.^7−9^ In ecDHFR, as in most WT enzymes with their physiological substrate, this reorganization results in a short and narrow distribution of DADs so that the effects of thermal fluctuations in the optimal tunneling ready state (TRS) are the same for all hydrogen isotopes. As a result, the intrinsic KIEs are temperature-independent.^18^ Disruption of the optimized TRS through site-directed mutagenesis, for example, results in DADs that are longer and more broadly distributed. Thus, temperature-dependent KIEs are observed for many mutants of the enzyme because at lower temperatures there is insufficient wavefunction overlap for effective tunneling of the heavier isotopes at the average DAD.

**Figure 1:**
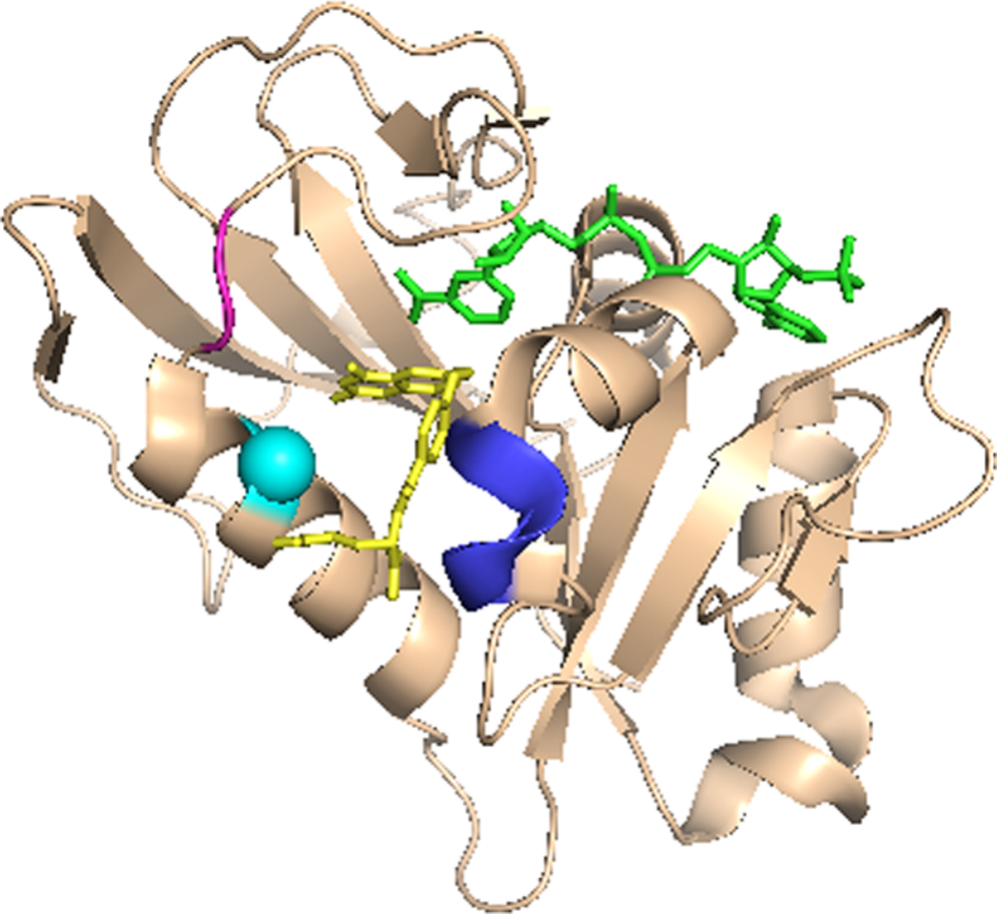
hsDHFR (PDB ID 4M6K) bound to NADP^+^ and folate. The ligands are shown as green (NADP^+^) and yellow (folate) sticks; hsDHFR residues corresponding to the PCEs described in^1^ are shown in cyan (F32), magenta (26-PPLR), and blue (62-PEKN).

Previous studies have linked the functional motions of DHFR to the evolution of the enzyme.^1,19,25,26^ Phylogenetic analysis of DHFR sequences in organisms ranging from bacteria to *Homo sapiens* DHFR (hsDHFR) identified three key sites that were predicted to influence the motions of the enzyme (Figure 1) and were denoted as phylogenetically coherent events (PCEs).^1^ The first PCE was a four-amino-acid insert (62-PEKN in hsDHFR) that appeared ∼797 million years ago and is conserved in higher organisms. A second PCE occurred ∼325 million years ago in the M20 loop of the enzyme and consists of a polyproline sequence (26-PPLR in hsDHFR).^1^ A third PCE is at the end of the M20 loop and corresponds to F32 in the human enzyme. The corresponding residue in the extensively studied *E. coli* enzyme is a leucine that first evolved to a methionine before becoming a phenylalanine ∼499 million years ago.

In their study, Benkovic et al. created and characterized several ecDHFR mutants to test their ideas about DHFR evolution and the PCEs that they identified and developed a predictive model of the forces that drove these events.^1^ A notable difference between the cellular environments of prokaryotic and eukaryotic cells is the intracellular pool of NADP^+^ and NADPH.^27^ Since the catalytic turnover of both prokaryotic and eukaryotic DHFRs is limited by product release,^28,29^ there was evolutionary pressure to respond to changing cellular environments while maintaining an efficient rate of enzyme-catalyzed hydride transfer. The PCE that introduced the polyproline sequence restricts the motion of the M20 loop that undergoes dramatic conformational changes during the turnover of ecDHFR.^30^ The restricted motion prevents significant product inhibition by NADP^+^ but also dramatically reduces both the efficiency of hydride transfer and the overall turnover rate of the enzyme.^1,19^ Thus, it appears that other PCEs compensate for the impaired hydride transfer resulting from the polyproline insertion in ecDHFR.

NMR relaxation measurements have shown that insertion of N23PP alone into ecDHFR restricts the ms dynamics of the enzyme.^30^ In addition, quantum-mechanics/molecular-mechanic (QM/MM) simulations demonstrated that the magnitudes of the conformational changes between the ground state and the transition state of the reaction of N23PP are increased relative to ecDHFR, which is indicative of greater motion of many residues across the enzyme to achieve the short DAD required for hydride transfer.^1,31^ This increase in the dynamics correlates with a decrease in the single turnover rates^1^ of the mutant and an increased temperature dependence of the KIEs^19^ compared to WT ecDHFR. Since there are no natural DHFRs containing only the 23PP insertion, a “humanized” form of ecDHFR was created through insertions of both the polyproline sequence and PEKN (N23PP-G51PEKN ecDHFR). QM/MM simulations of this variant found that the dynamics were restored to those found in WT ecDHFR.^1^ In addition, both the single turnover rates and the temperature dependence of the KIEs were identical to WT ecDHFR.^1,19^ Since the “rescuing” PEKN insertion occurred first in the evolution of the enzyme, it appears that the protein motions in DHFR were preserved through evolution and likely play a critical role in enzyme catalysis.

The network of coupled residues that have been extensively studied in the *E. coli* enzyme^20,21,23^ has also been shown to contribute to hydride transfer in hsDHFR.^25^ In the bacterial enzyme, residues M42 and G121, which are distal to the active site, function synergistically to afford an efficient hydride transfer. Sequence alignments show that the equivalent residues in hsDHFR are M53 and S145, respectively. Applying a similar approach of site-directed mutagenesis and investigation of the temperature dependence of the intrinsic KIEs on hydride transfer catalyzed by the variants of the human enzyme showed a similar synergistic effect of these residues. It thus appears that the network of coupled residues in DHFR catalysis is also conserved in evolution. This led us to hypothesize that the PCEs act synergistically to conserve the properties of the enzyme at the TRS, especially the short and narrow distribution of DADs, during evolution.

The current study tests similar effects of PCEs as the aforementioned studies have done in ecDHFR, except we now focus on the effects of backward mutations from hsDHFR. Specifically, we examine the effects of these PCEs on the temperature dependence of the KIE in several site-directed mutants of the human enzyme in which we mutate residues of the human enzyme back to a sequence like that found in the ecDHFR. First, F32 in hsDHFR is sequentially mutated back toward the bacterial enzyme by creating an F32M variant to mimic the form that first appeared ∼797 million years ago and then the F32L variant to mimic ecDHFR. We also study deletion mutants of the PCE insertions identified by Benkovic et al., 26PP and 62-PEKN. Finally, we combine all of these to create a “bacterialized” version of the human enzyme. Each of the residues studied here is highlighted in the X-ray structure of the enzyme with NADP^+^ and folate bound (Figure 1), and the variants are summarized in Table 1, which also notes which PCE each variant is designed to assess. These studies test the evolutionary importance of 32F, 26PP, and 62-PEKN in maintaining a short and narrow distribution of DADs for effective hydride tunneling. If the effects of these residues on the catalyzed hydride transfer are preserved through evolution, it supports the general idea that evolution will retain optimal TRS properties for the catalyzed H-transfer reactions even when the chemistry is not rate limiting.

**Table 1:** Summary of hsDHFR Variants Characterized

hsDHFR variants	PCE^a^	divergence (mya)^b^	region of the enzyme
Δ62-PEKN	62-PEKN	∼797	distal to M20 loop
F32M, F32L	F32	∼499	at base of M20 loop
Δ26-PPLR-Δ62-PEKN	26-PPLR	∼325	within M20 loop
F32L-PP26N-PEKN62G	all PCE		

a Phylogenetically coherent events identified in ref (32).

b Time of divergence in millions of years (mya).

## Materials and Methods

### Materials

All chemicals are reagent grade and purchased from Sigma-Aldrich (St. Louis, MO), unless otherwise mentioned. [Carbonyl-^14^C]-nicotinamide was from Moravek, while [^3^H]-sodium borohydride was from Perkin-Elmer. Glucose dehydrogenase from *Bacillus megaterium* is from MP Biomedical. [Carbonyl-^14^C]-NADPH, 4R-[carbonyl -^14^C, 4-^2^H]-NADPH, 4R-^3^H-NADPH, and DHF were synthesized and stored according to previously published protocols.^33−39^

### Preparation of Mutant Variants

All mutant variants were constructed using the Agilent Stratagene QuickChange site-directed mutagenesis kit using the manufacturer’s instructions. The F32L, F32M, PP26N, and the Δ62-PEKN deletion mutants were derived from the hsDHFR WT plasmid. We constructed the Δ26-PPLR-Δ62-PEKN and the F32L-PP26N-PEKN62G mutants through sequential reactions using different mutant plasmids. For the double-deletion mutant (Δ26-PPLR-Δ62-PEKN), we performed site-directed mutagenesis using the Δ62-PEKN plasmid derived from the WT. Similarly, we constructed the triple mutant (F32L-PP26N-PEKN62G) by replacing 26PP with N using the PP26N primers and then using the resulting plasmid for a subsequent round of mutagenesis in which G replaces 62-PEKN. All mutagenic primers used to generate the hsDHFR variants were from Integrated DNA Technologies (Coralville, IA) and their sequences are listed in Table S1. The PCR products were transformed into *E. coli* DH5α cells. We extracted plasmids from overnight cultures to confirm the sequences by automated DNA sequencing at the University of Iowa DNA Core Facility. All hsDHFR variants were expressed and purified following published protocols described previously.^2^

### Competitive Intrinsic KIE Measurements

We measured intrinsic KIEs as described previously for human and ecDHFR.^2,18^ In summary, reacting the protium- or tritium-labeled NADPH at the 4R position with unlabeled DHF at the desired temperature at pH 9.0 in 50 mM MTEN buffer (50 mM MES, 25 mM Tris, 25 mM ethanolamine, and 100 mM sodium chloride) yielded a measure of the H/T KIE. The carbonyl carbon on the nicotinamide ring of the cofactor was labeled with ^14^C to serve as a tracer for the conversion of protiated NADPH to product (NADP^+^). An excess of methotrexate quenched the reactions at different time intervals, and we stored the reaction mixture on dry ice. Oxygen bubbled into the quenched reaction mixture ensured that THF was completely oxidized before high-performance liquid chromatography (HPLC) analysis. D/T experiments followed the same protocol but with deuterium at the 4R position. The depletion of tritium in the substrate as a function of fractional conversion yielded the observed KIE on the second-order rate constant *k*_cat_/*K*_M_. Intrinsic KIEs were then extracted from the observed H/T and D/T KIEs using the Northrop equation (eq 1)^40^
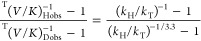
1In eq 1, ^T^(*V*/*K*)_Hobs_ and ^T^(*V*/*K*)_Dobs_ are the observed H/T and D/T KIEs, respectively, and *k*_H_/*k*_T_ is the intrinsic H/T KIE. The isotope effects on the activation parameters for the intrinsic KIEs are calculated by a nonlinear fit of the data to the Arrhenius equation for intrinsic KIEs (eq 2)
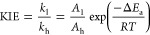
2where *k*_l_ and *k*_h_ are the rate constants for light and heavy isotopes, respectively, *A*_l_/*A*_h_ is the isotope effect on the Arrhenius pre-exponential factor, Δ*E*_a_ is the difference in energy of activation between the two isotopologues, *R* is the gas constant, and *T* is the absolute temperature.

### Steady-State Kinetics

The steady-state kinetic parameters of the deletion mutants were determined in 50 mM MTEN buffer at pH 7.65 and 25 °C for comparison to the WT enzyme.^2^ We were unable to measure the single turnover rate of hsDHFR due to a high rate constant even at 4 °C leading to the reaction occurring within the dead time of the stopped flow instrument (∼2 ms). Thus, the effects of the deletion mutants of hsDHFR residues were limited to the steady-state turnover of the enzyme. In short, initial rates were determined with ∼50 nM of the enzyme by following the decrease in absorbance of NADPH at 340 nm, which occurred when DHF and NADPH convert to THF and NADP^+^. The kinetic parameters with DHF were determined using the 0.1–1.0 μM substrate and those with NADPH were determined using 0.3–1.5 μM nicotinamide. For each set of experiments, the second substrate was kept constant at a saturating concentration of 100 μM. Plots of the initial rate as a function of the substrate concentration were fit to the Michaelis–Menten equation for one substrate to determine the kinetic parameters.

## Results and Discussion

The first PCE investigated here is F32, which was originally an L (L28 in ecDHFR). This PCE occurred after the PEKN insertion but before the development of PPLR. The transition away from the L residue in ecDHFR occurs essentially simultaneously with the insertion of PEKX that ultimately becomes PEKN in hsDHFR. It is reasonable to speculate that mutation of L to F32 occurred as a way of restricting the motion of the M20 loop to regulate the binding of NADPH and release of NADP^+^ before the more substantial PPLR insertion developed. To test this idea, we use site-directed mutagenesis of hsDHFR and measure the intrinsic KIEs on hydride transfer. Following the evolutionary process backward, we first mutate F32 in hsDHFR to M to mimic the transitional form of DHFR and then to L as in ecDHFR.

**Figure 2:**
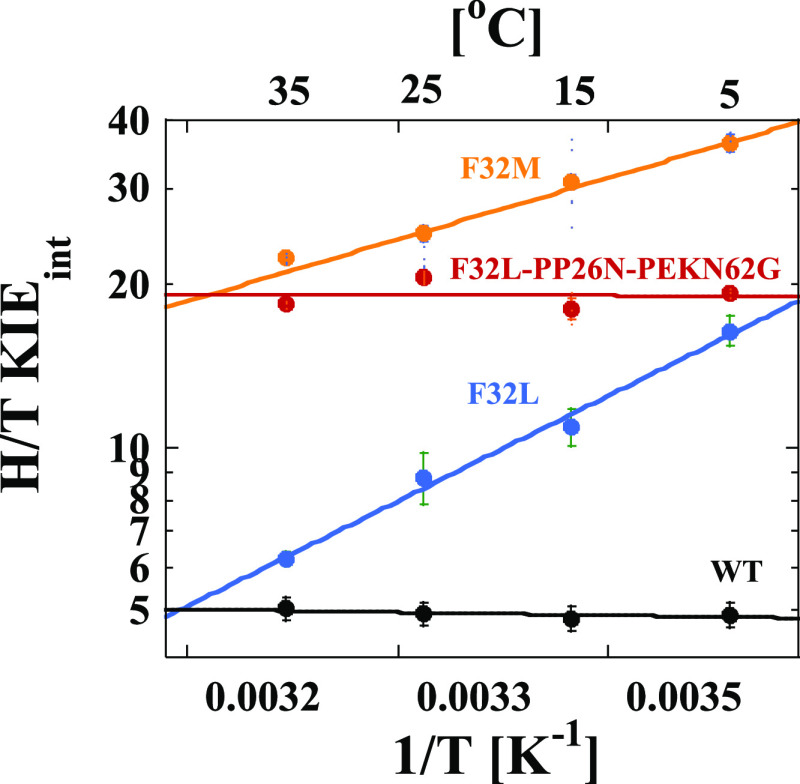
Arrhenius plots of the H/T KIEs for each hsDHFR PCE at pH 9.0. The data points are the average of at least 5 independent measurements with their standard deviations and the lines are nonlinear fits of all measured KIEs to eq 2. The lines are for WT hsDHFR (black),^2^ F32L (blue), F3M (orange), and F32L-PP26N-PEKN62G (red).

Figure 2 shows the temperature dependence of the intrinsic KIEs on hydride transfer for the F32 variants of hsDHFR. Replacing F32 with M to resemble a transitional DHFR has a large effect on both the magnitude and temperature dependence of the intrinsic KIE for the enzymatic reaction. The size of the KIE is higher than any hsDHFR measured previously. Importantly, the temperature dependence of the KIE (Δ*E*_a(T-H)_) is substantially larger than for the WT enzyme with a value of 2.8 ± 0.2 kcal/mol. Fitting the data to the phenomenological activated tunneling model^41^ yields an average DAD at the TRS of 3.20 ± 0.01 Å (Table 2). Note that this distance is the average DAD, not the DAD at which tunneling occurs. Tunneling can only occur at shorter DADs, which are thermally accessible in the distribution but rare. The significant temperature dependence of the KIE reflects the thermal sampling of the DAD distribution within the active site that is required to reach a DAD that is suitable for hydride tunneling. For F32L, the temperature dependence is even larger, with a Δ*E*_a(T-H)_ value of 6.5 ± 0.3 kcal/mol. This corresponds to an average DAD at the TRS of 3.48 ± 0.02 Å.

**Table 2:** Comparative Isotope Effects for hsDHFR Mutants at pH 9.0

hsDHFR variant	*A*_H_/*A*_T_	Δ*E*_a(T-H)_ kcal/mol	DAD_avg_^a^ (Å)
wild-type hsDHFR	6.6 ± 0.8	–0.1 ± 0.1	3.05 ± 0.01
F32M	0.2 ± 0.1	2.8 ± 0.2	3.20 ± 0.01
F32L	0.1 ± 0.1	6.5 ± 0.3	3.48 ± 0.02
F32L-PP26N-PEKN62G	19.3 ± 6.7	–0.01 ± 0.2	3.10 ± 0.01
Δ62-PEKN	3.1 ± 0.3	0.5 ± 0.1	3.10 ± 0.01
Δ26-PPLR-Δ62-PEKN	5.2 ± 0.5	0.1 ± 0.1	3.05 ± 0.01

a From a fit of the data to the model described in ref (41).

Two additional PCEs occurred along with evolution to F32. To fully mimic ecDHFR using the human enzyme as a starting point, we created a triple mutant to account for all three PCEs identified by Benkovic et al.^1^ This triple mutant replaces F32 with L and removes both the rigidifying PP mutation through the replacement of 26PP with N and the flexibility inducing PEKN insertion by replacing 62-PEKN with G. We identify this triple mutant as F32L-PP26N-PEKN62G. Remarkably, the triple mutant is not only still active but also, based on the temperature-independent KIE values in Figure 2, able to achieve a narrow distribution of DADs at the TRS similar to both hsDHFR and ecDHFR. The Δ*E*_a(T-H)_ value is −0.01 ± 0.05 kcal/mol and an average DAD of 3.09 ± 0.01 Å, which is strikingly similar to the value for WT hsDHFR. Notably, however, the average KIE is ∼2–3 times larger than that for ecDHFR, which reflects the fact that while the distribution of DADs is narrow and close to that for the more evolved enzyme, it is not fully optimized at the shortest possible DAD that would be expected for a more evolved enzyme.

After identifying the PCEs of DHFR evolution, Benkovic et al. explored these in the bacterial enzyme through insertion of both PEKN and PP into the ecDHFR.^1^ Insertion of PP alone was found to dramatically impair both the steady-state and single turnover rates of the enzyme. These insertions were also later shown to affect the temperature dependence of the intrinsic KIEs on the reaction.^19^ The effect of the PP insertion was mitigated by also inserting PEKN in a manner similar to what we find for the triple mutant, the F32L-PP26N-PEKN62G hsDHFR variant described above. To test the effects of the 62-PEKN and the 26-PPLR PCEs in hsDHFR, we construct single and double-deletion mutants of these sites and measure the temperature dependence of the intrinsic KIEs. If our current hypotheses described above for the role of these insertions are correct, we would expect that these deletion mutants would behave in a fashion similar to the ecDHFR insertion variants. In addition, since the single turnover rate of WT hsDHFR cannot be measured due to a rate constant that is too fast for the dead time of the apparatus,^2^ we measure the steady-state turnover of the deletion mutants with both DHF and NADPH as substrates.

**Figure 3:**
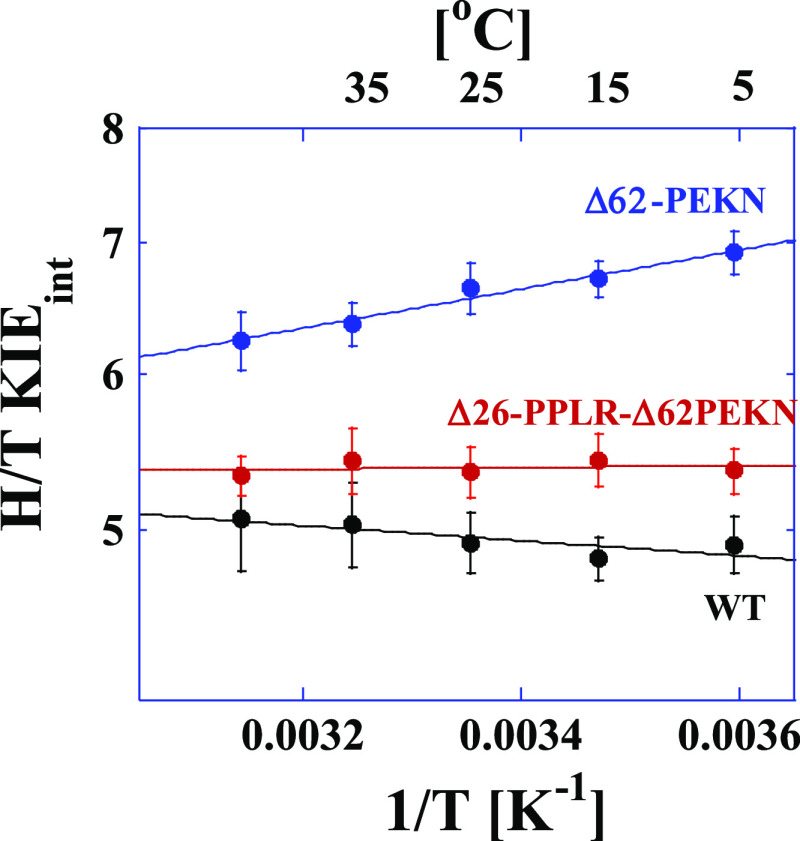
Arrhenius plots of the H/T KIEs for hsDHFR and the deletion mutant variants at pH 9.0. The data points are the average of at least 5 independent measurements with their standard deviations and the lines are nonlinear fits of all measured KIEs to eq 2. The lines are for WT hsDHFR (black),^2^ Δ62-PEKN (blue), and Δ26-PPLR-Δ62-PEKN (red).

The first deletion variant we consider removes the first PCE in the hsDHFR evolution (62-PEKN; blue in Figure 3) but leaves the PPLR sequence intact. Deletion of 62-PEKN results in KIEs that are both larger and more temperature-dependent compared to the WT hsDHFR enzyme. A fit of the data for the Δ62-PEKN mutant to the Arrhenius equation yields a Δ*E*_a(T-H)_ of 0.5 ± 0.1 kcal/mol (Table 1). Within the context of the activated tunneling model,^41^ this result suggests that the mutant enzyme is unable to achieve the short and narrow distribution of DADs required for efficient hydride tunneling with an average DAD of 3.10 Å. This enzyme retains the polyproline sequence that restricts M20 loop movement to control ligand flux but lacks the compensatory effects that the PEKN insertion provides. The deletion of PEKN alone also significantly reduces the overall turnover rate and the catalytic efficiency of the enzyme with both NADPH and DHF as substrates (Table 3). Similar effects were observed for the N23PP mutant of ecDHFR^1^ where restricting the M20 loop alone in the enzyme resulted in a disrupted TRS that manifests in impaired steady-state kinetic parameters^1^ and increased temperature dependence of the intrinsic KIE.^19^

**Table 3:** Steady-State Kinetic Parameters of Wild-Type and Mutant hsDHFR at pH 7.65 and 25 °C^a^^,^^b^

	wild-type^c^	Δ26-PPLR-Δ62-PEKN	Δ62-PEKN
*k*_cat_^DHF^, (s^–1^)	14.03 ± 0.02	11.28 ± 0.26	1.72 ± 0.04
*K*_m_^DHF^, (μM)	0.29 ± 0.05	0.40 ± 0.04	0.59 ± 0.06
(*k*_cat_/*K*_m_)^DHF^ × 10^7^ (M^–1^ s^–1^)	4.84 ± 0.83	2.82 ± 0.29	0.29 ± 0.03
*k*_cat_^NADPH^, (s^–1^)	14.10 ± 0.01	11.23 ± 0.13	1.73 ± 0.04
*K*_m_^NADPH^, (μM)	0.36 ± 0.05	0.54 ± 0.03	0.77 ± 0.08
(*k*_cat_/*K*_m_)^NADPH^ × 10^7^ (M^–1^ s^–1^)	3.92 ± 0.54	2.08 ± 0.12	0.22 ± 0.02

a *k*_cat_^DHF^ and *K*_m_^DHF^ are the turnover number and Michaelis constant with DHF as the substrate, measured in the presence of 100 μM NADPH and varying concentrations of DHF.

b *k*_cat_^NADPH^ and *K*_m_^NADPH^ are the turnover number and Michaelis constant with NADPH as the substrate, measured in the presence of 100 μM DHF and varying concentrations of NADPH.

c From ref (2).

The PCE that introduced flexibility in DHFR occurred incredibly early in the evolution of the enzyme (PEKN). This flexibility likely allowed for the subsequent rigidification that the polyproline sequence produces to modulate the binding and release of NADPH and NADP^+^. Consistent with the results for the F32L-PP26N-PEKN62G described above, the double-deletion variant that removes both 62-PEKN and 26-PPLR from hsDHFR restores the ability of the enzyme to achieve an optimal TRS for hydride transfer as is evident from the temperature-independent KIE (red line in Figure 3; Δ*E*_a(T-H)_ = 0.1 ± 0.1 kcal/mol in Table 1). As shown in Table 3, deletion of 26-PPLR and 62-PEKN largely restores the overall rate of catalytic turnover as well as the catalytic efficiencies with both NADPH and DHF as substrates. Finally, fitting the data for the Δ26-PPLR-Δ62-PEKN variant to the phenomenological activated tunneling model^41^ gives an average DAD at the TRS that is identical to that of the WT (3.05 Å; Table 1).

The results of these variants show that the changes caused by the deletion of the PEKN and PPLR PCEs follow precisely the expected trend based on the previous studies of the insertions of these sequences in ecDHFR. That result is significant because it corroborates the model suggested by the previous studies regarding the effects of these sequences on the substrate binding and the catalyzed reaction. Although that outcome may seem obvious and expected based on the prior studies, it is nevertheless impressive because the effects of evolutionary changes are not always so consistent with predictions.

Our results provide strong support for the model that Benkovic et al.^1^ put forth to understand the physical effects of the PCEs that occur between ecDHFR and hsDHFR. The changing ligand concentrations in the intracellular environments of prokaryotic and eukaryotic cells placed evolutionary pressure on DHFR whose activity is essential for cellular function. Large-scale conformational changes of the M20 loop in ecDHFR allow the enzyme to achieve the short and narrow distribution of DADs at the TRS for efficient hydride transfer, but it also makes the enzyme susceptible to inhibition by NADP^+^.^1^ Such inhibition reduces both the turnover rate and catalytic efficiency of the enzyme, which would be catastrophic to cellular metabolism in the presence of higher concentrations of NADP^+^. This sequence suggests that the enzyme may have evolved initially to increase the flexibility of the entire protein through insertion of the PEKN sequence. The subsequent PP insertion and mutations at L28 could then restrict the motions of the M20 loop while maintaining enough flexibility in the active site to achieve the short and narrow distribution of DADs required for efficient hydride transfer and avoiding product inhibition. The evolutionary studies of DHFR in this and previous work suggest that a delicate balance is required for efficient catalysis in different cellular environments.

## Conclusions

We use site-directed mutagenesis and measurements of intrinsic KIEs to study the PCEs identified by Benkovic et al.^1^ in the human DHFR enzyme. In each case, we prepare mutant variants that remove the PCEs from the hsDHFR sequence to bacterialize the enzyme. We find that single mutations or deletions of some of the key PCEs perturb the distribution of DADs at the TRS leading to longer average DADs with a wider distribution that results in thermal sampling giving rise to a temperature-dependent KIE for the catalyzed hydride transfer. Preparing either the triple mutant (F32L-PP26N-PEKN62G) or the double-deletion variant (Δ26-PPLR-Δ62-PEKN), however, effectively rescues the catalytic properties of the enzyme resulting in a temperature-independent KIE suggesting a narrow DAD distribution centered at an average that is only slightly larger than that for the WT hsDHFR, Thus, the bacterialized hsDHFR has properties that are very similar to those for WT ecDHFR. These results are consistent with prior studies of the humanized ecDHFR.^19^ In that study, the PP insertion into ecDHFR altered the DAD distribution leading to a temperature-dependent KIE. In contrast, the double insertion mutant with both the PP and PEKN sequences added to ecDHFR exhibited a temperature-independent KIE indicative of recovery of a DAD distribution that was similar to that for the WT ecDHFR. Thus, our results support the physical model for these PCEs that was first hypothesized by Benkovic et al., and they are consistent with prior studies of ecDHFR variants that mimic the same evolutionary PCEs.

## Supporting Information Available

Tables showing the primers used in this study and the observed and intrinsic H/T and D/T KIEs for each variant studied are provided (PDF)

## Accession Codes

DYR-HUMAN, P00374; DYR_ECOLI, P0ABQ4.

## Abbreviations

(DHFR): dihydrofolate reductase
(NADPH): reduced nicotinamide adenine diphosphate
(DHF): 7,8-dihydrofolate
(THF): 5,6,7,8-tetrahydrofolate
(KIEs): kinetic isotope effects
(WT): wild-type
(ecDHFR): *Escherichia coli* dihydrofolate reductase
(hsDHFR): human dihydrofolate reductase
(DAD): donor–acceptor distance
(TRS): tunneling ready state
(PCEs): phylogenetically coherent events
