# Supplementary material for: Evolution of Optimized Hydride Transfer Reaction and Overall Enzyme Turnover in Human Dihydrofolate Reductase
Source: Biochemistry. 2021 Dec 7;60(50):3822–8. doi: 10.1021/acs.biochem.1c00558 (PMC8697555; doi:10.1021/acs.biochem.1c00558)
Supplement: Supplementary file 1 — bi1c00558_si_001.pdf [file bi1c00558_si_001.pdf]

Evolution of Optimized Hydride Transfer Reaction and Overall Enzyme Turnover in Human Dihydrofolate Reductase

Author List: Jiayue Li,[1] Jennifer Lin,[1] Amnon Kohen,[1] Priyanka Singh,[1] Kevin Francis[*2], Christopher M. Cheatum[*1]

Institution Addresses:

[1] Department of Chemistry, University of Iowa, Iowa City, IA 52242

[2] Texas A&M University-Kingsville, Kingsville. TX, 78363

Author Information Notes:

* Authors to whom correspondence should be addressed:

Email: christopher-cheatum@uiowa.edu Phone: +1 319 353 0379

kevin.francis@tamuk.edu Phone: + 1 361 595 2923

Table S1: Primers Used in This Study

mutant	primer sequence
F32M	5’-GGCCACCGCTCAGGAATGAAATGAGATATTTCCAGAGAATGAC-3’
	5’-GTCATTCTCTGGAAATATCTCATTTCATTCCTGAGCGGTGGCC-3’
F32L	5’-CCACCGCTCAGGAATGAATTGAGATATTTCCAGAGAATGAC-3’
	5’-GTCATTCTCTGGAAATATCTCAATTCATTCCTGAGCGGTGG-3’
PP26N	5’-GACCTGCCCTGGCTCAGGAATGAATTC-3’
	5’-GAATTCATTCCTGAGCCAGGGCAGGTC-3’
PEKN62G	5' CTGGTTCTCCATTCGAAGAATTAATTTAGTTC-3'
	5' GAACTAAATTAATTCTTCGAATGGAGAACCAG 3'
Δ62-PEKN	5' GGTTCTCCATTCGACCTTTAAAGGG 3'
	5' CCCTTTAAAGGTCGAATGGAGAACC-3'
Δ26-PPLR-Δ62–PEKN	5' ACCTGCCCTGGCCAGAATTCAGATA 3'
	5' TATCTGAATTCTGGCCAGGGCAGGT 3'

Table S2: Intrinsic and Observed KIEs of F32M hsDHFR at pH 9.0[a]

Temp. ˚C	Observed H/T KIE	Observed D/T KIE	Intrinsic H/T KIE
5	1.89 ± 0.04	1.48 ± 0.01	36.38 ± 1.41
15	2.29 ± 0.04	1.60 ± 0.01	31.06 ± 0.90
25	1.85 ± 0.03	1.43 ± 0.01	24.78 ± 0.90
35	2.66 ± 0.06	1.70 ± 0.02	22.29 ± 0.41

[a] Observed KIEs were measured in 50 mM METN buffer (50 mM MES, 25 mM Tris, 25 mM ethanolamine, and 100 mM NaCl). The values represent at least 5 independent measurements with their standard deviation.

Table S3: Intrinsic and Observed KIEs of F32L hsDHFR at pH 9.0[a]

Temp. ˚C	Observed H/T KIE	Observed D/T KIE	Intrinsic H/T KIE
5	1.91 ± 0.03	1.41 ± 0.04	19.06 ± 1.05
15	2.00 ± 0.05	1.39 ± 0.01	11.24 ± 0.82
25	1.93 ± 0.14	1.36 ± 0.06	8.44 ± 1.91
35	1.90 ± 0.01	1.31 ± 0.01	5.93 ± 0.19

[a] Observed KIEs were measured in 50 mM METN buffer (50 mM MES, 25 mM Tris, 25 mM ethanolamine, and 100 mM NaCl). The values represent at least 5 independent measurements with their standard deviation.

Table S4: Intrinsic and Observed KIEs of F32L-PP26N-PEKN62G hDHFR at pH 9.0[a]

Temp. ˚C	Observed H/T KIE	Observed D/T KIE	Intrinsic H/T KIE
5	1.97 ± 0.02	1.44 ± 0.01	19.31 ± 0.37
15	3.01 ± 0.05	1.70 ± 0.01	18.09 ± 0.93
25	3.10 ± 0.02	1.75 ± 0.01	20.29 ± 0.26
35	3.42 ± 0.02	1.78 ± 0.01	18.38 ± 0.21

[a] Observed KIEs were measured in 50 mM METN buffer (50 mM MES, 25 mM Tris, 25 mM ethanolamine, and 100 mM NaCl). The values represent at least 5 independent measurements with their standard deviation.

Table S5: Intrinsic and Observed KIEs of Δ62-PEKN hDHFR at pH 9.0[a]

Temp. ˚C	Observed H/T KIE	Observed D/T KIE	Intrinsic H/T KIE
5	4.13 ± 0.02	1.64 ± 0.02	6.92 ± 0.18
15	3.97 ± 0.04	1.62 ± 0.01	6.71 ± 0.14
25	3.77 ± 0.03	1.60 ± 0.03	6.63 ± 0.20
35	3.53 ± 0.02	1.57 ± 0.01	6.37 ± 0.16
45	3.40 ± 0.03	1.55 ± 0.02	6.24 ± 0.21

[a] Observed KIEs were measured in 50 mM METN buffer (50 mM MES, 25 mM Tris, 25 mM ethanolamine, and 100 mM NaCl). The values represent at least 5 independent measurements with their standard deviation.

Table S6: Intrinsic and Observed KIEs of Δ26-PPLR-Δ62-PEKN hDHFR at pH 9.0[a]

Temp. ˚C	Observed H/T KIE	Observed D/T KIE	Intrinsic H/T KIE
5	3.96 ± 0.02	1.57 ± 0.01	5.36 ± 0.14
15	3.60 ± 0.02	1.54 ± 0.01	5.43 ± 0.17
25	3.39 ± 0.01	1.52 ± 0.01	5.35 ± 0.15
35	3.17 ± 0.03	1.50 ± 0.02	5.43 ± 0.21
45	3.02 ± 0.01	1.48 ± 0.01	5.33 ± 0.12

[a] Observed KIEs were measured in 50 mM METN buffer (50 mM MES, 25 mM Tris, 25 mM ethanolamine, and 100 mM NaCl). The values represent at least 5 independent measurements with their standard deviation.
